# Does ICU admission dysphagia independently contribute to delirium risk in ischemic stroke patients? Results from a cohort study

**DOI:** 10.1186/s12888-024-05520-w

**Published:** 2024-01-23

**Authors:** Hongtao Cheng, Simeng Song, Yonglan Tang, Shiqi Yuan, Xiaxuan Huang, Yitong Ling, Zichen Wang, Xiaoying Tian, Jun Lyu

**Affiliations:** 1 School of Nursing, Jinan University, Guangzhou, China; 2 Department of Clinical Research, The First Affiliated Hospital of Jinan University, Guangzhou, China; 3 Department of Neurology, The First Affiliated Hospital of Jinan University, Guangzhou, China; 4 Guangdong Provincial Key Laboratory of Traditional Chinese Medicine Informatization, Guangzhou, China

**Keywords:** Dysphagia, Ischemic stroke, Delirium, Intensive care unit, Cohort study

## Abstract

**Background:**

Delirium is prevalent in ischemic stroke patients, particularly those in the intensive care unit (ICU), and it poses a significant burden on patients and caregivers, leading to increased mortality rates, prolonged hospital stays, and impaired cognitive function. Dysphagia, a common symptom in critically ill patients with ischemic stroke, further complicates their condition. However, the association between dysphagia and delirium in this context remains unclear. The objective of this study was to investigate the correlation between dysphagia and delirium in ICU patients with ischemic stroke.

**Methods:**

A retrospective analysis was conducted on adult patients diagnosed with ischemic stroke at a medical center in Boston. Ischemic stroke cases were identified using the ninth and tenth revisions of the International Classification of Diseases. Dysphagia was defined as a positive bedside swallowing screen performed by medical staff on the day of ICU admission, while delirium was assessed using the ICU Confusion Assessment Method and review of nursing notes. Logistic regression models were used to explore the association between dysphagia and delirium. Causal mediation analysis was employed to identify potential mediating variables.

**Results:**

The study comprised 1838 participants, with a median age of approximately 70 years, and 50.5% were female. Among the total study population, the prevalence of delirium was 43.4%, with a higher prevalence observed in the dysphagia group (60.7% vs. 40.8%, *p* < 0.001) compared to the non-dysphagia group. After adjusting for confounding factors including age, sex, race, dementia, depression, sedative medications, history of falls, visual or hearing deficit, sequential organ failure score, and Glasgow coma score, multifactorial logistic regression analysis demonstrated a significant association between dysphagia and an increased likelihood of delirium (odds ratio [OR]: 1.48; 95% confidence interval [CI]: 1.07–2.05; *p* = 0.018; E-value = 1.73). Causal mediation analysis revealed that serum albumin levels partially mediated the association between dysphagia and delirium in critically ill patients with ischemic stroke (average causal mediated effect [ACME]: 0.02, 95% CI: 0.01 to 0.03; *p* < 0.001).

**Conclusion:**

ICU admission dysphagia may independently contribute to the risk of delirium in patients with ischemic stroke. Early identification and intervention in ischemic stroke patients with dysphagia may help mitigate the risk of delirium and improve patient prognosis.

**Supplementary Information:**

The online version contains supplementary material available at 10.1186/s12888-024-05520-w.

## Introduction

Stroke is the second leading cause of death and the leading cause of disability worldwide [1]. Approximately 80% of strokes are ischemic in nature, and nearly 15–20% of people admitted to stroke units require treatment in intensive care units (ICU) [2, 3]. Worryingly, the long-term mortality rate in the ICU is a staggering 66%, while only 8–14% of patients have a favorable prognosis and survive [4]. In addition, ischemic stroke is associated with a wide range of complications, including dysphagia, dysarthria, aphasia, stroke-associated pneumonia, post-stroke depression and post-stroke delirium [1, 5]. These complications pose a significant threat to the well-being and longevity of patients and contribute significantly to the global economic burden of stroke [6]. In the United States, the estimated average lifetime medical cost per ischemic stroke patient is a staggering $140,000 [7]. Despite recent declines in stroke incidence, the aging of the world’s population, combined with the accumulation of risk factors, has resulted in an increased lifetime risk of stroke [6]. There is therefore an urgent need to develop and implement stroke-specific early prevention and care strategies to improve outcomes, reduce the incidence of stroke and reduce the risk of associated complications.

Complications have a well-documented propensity to worsen disease prognosis and consistently undermine the quality of life of people coping with acute and critical illness [8]. Stroke-associated dysphagia and delirium are strongly associated with prolonged hospitalization, increased in-hospital mortality, and poor functional prognosis [8–10]. Prioritizing the prevention and treatment of these conditions is therefore of paramount importance, as they have a significant impact on the quality of life and overall prognosis of stroke patients. Studies [5, 11] have shown that the incidence of dysphagia after stroke ranged from 34.11 to 80.05%, while the incidence of delirium after stroke ranged from 10 to 48%. Dysphagia not only increased the risk of aspiration pneumonia and prolonged hospital stay in stroke patients, but also emerged as an independent risk factor for malnutrition, impaired functional recovery, and post-stroke depression [5]. Notably, there was a positive association between the severity of dysphagia and delirium. In geriatric syndromes, delirium was found to be a risk factor for dysphagia, effectively doubling the likelihood of developing swallowing difficulties [12]. In one study, the prevalence of concurrent delirium after dysphagia in elderly patients ranged from 22 to 59.4% [13]. However, these findings showed considerable variability due to factors such as heterogeneity within the study population, variations in screening and diagnosis of dysphagia, sample size, and other confounding factors. Currently, there is insufficient conclusive evidence to support an association between dysphagia and delirium in patients with ischemic stroke during critical illness. Early assessment of dysphagia in patients with delirium should facilitate early intervention with targeted rehabilitation therapy for dysphagia. Previous studies had identified associations between dysphagia and variables such as age, sex, neurological disease, cardiac disease, low serum albumin levels, and the presence of delirium after hip fracture surgery [14]. In particular, dysphagia significantly affected food intake in stroke patients, resulting in weight loss and decreased albumin levels, especially in critically ill patients [15]. Meanwhile, malnutrition emerged as a major risk factor for delirium in elderly patients, with serum albumin being an important predictor of delirium [16]. Unfortunately, insufficient attention has been paid to dysphagia in patients with ischemic stroke during critical illness and their subsequent risk of developing delirium in the ICU. Furthermore, only a limited number of studies had examined potential mediating variables contributing to the association between these two conditions. Investigating these mediating factors held promise for improving management and clinical interventions for people with ischemic stroke during critical illness.

The main objective of this study was to investigate the association between dysphagia and delirium in patients with ischemic stroke during critical illness. In addition, the study aimed to investigate the potential mediating role of serum albumin levels in the association between dysphagia and delirium in these patients, with the aim of providing evidence to inform the clinical prevention, management and care of dysphagia after ischemic stroke. Based on the expertise of clinical neurologists and the previous literature [17, 18], the study hypothesized that dysphagia would independently increase the risk of delirium in patients with ischemic stroke.

## Methods

### **Data sources and ethical considerations**

Data for this study were collected from the Medical Information Mart for Intensive Care (MIMIC-IV) database, version 2.0, which served as the basis for conducting an observational cohort study [19, 20]. The MIMIC-IV database is a large and publicly available intensive care database containing electronic health records (EHR) from the Beth Israel Deaconess Medical Center in Boston, Massachusetts. It contains comprehensive medical and nursing information for a significant number of patients admitted to the ICU between 2008 and 2019. These records were regularly documented either by the MetaVision bedside monitoring system or by critical care nurses. The MIMIC-IV database is a valuable resource for critical care research, enabling the study of various clinical medical care issues and supporting informed medical decision making. One of the authors of this study, Hongtao Cheng, accessed the database after completing the required training and certification (record ID: 45,369,280) (eFig. 1). As our study had no direct impact on clinical care and all protected health information was de-identified to protect patient privacy, individual patient consent was not required. The study adhered to the tenets of the Declaration of Helsinki.

### Patients

**Fig. 1:**
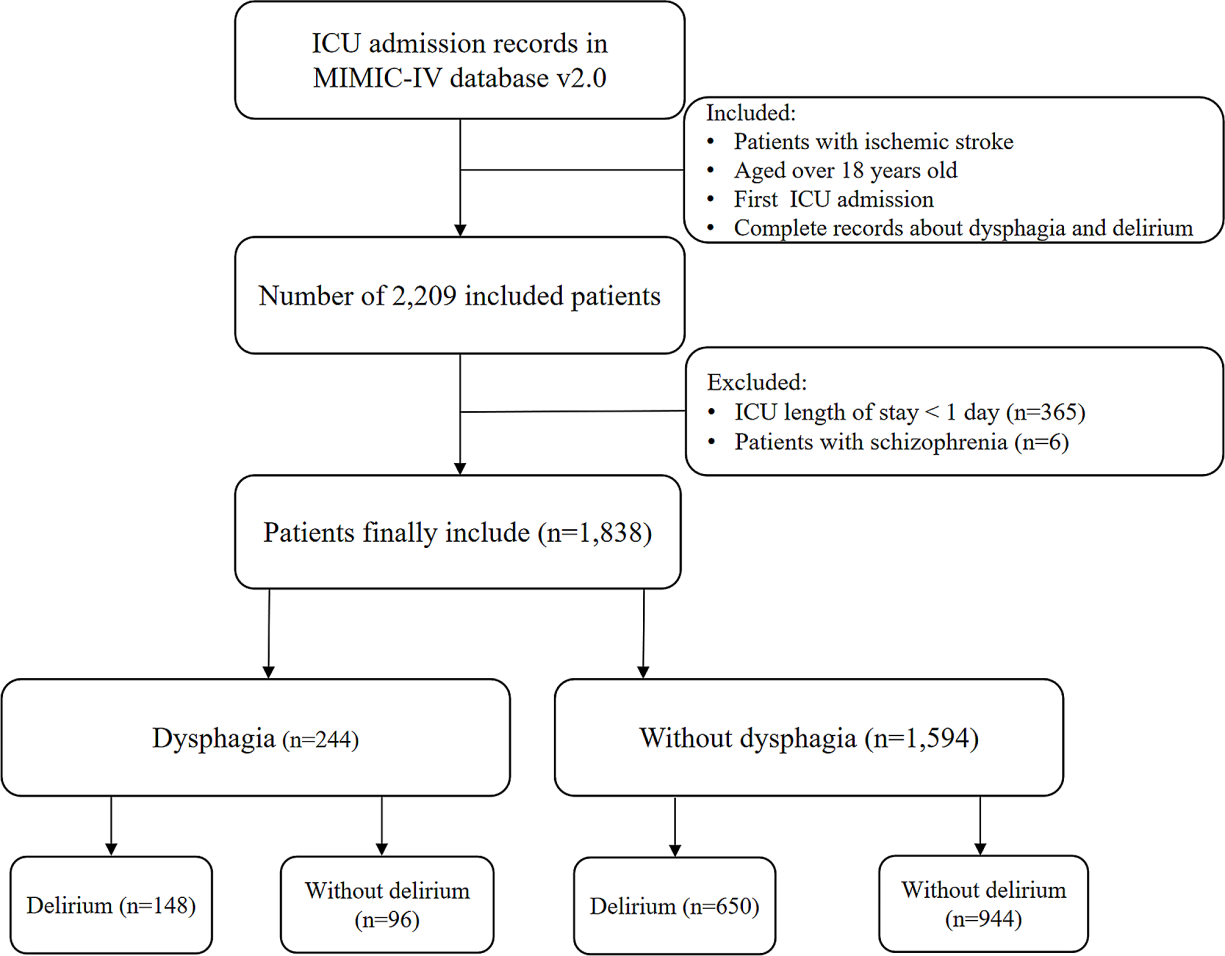
Inclusion and exclusion flowchart of the study. Abbreviations: ICU: Intensive Care Unit; MIMIC-IV: Medical Information Mart for Intensive Care IV

The inclusion criteria for this study included patients who met the following conditions: (1) diagnosed ischemic stroke, (2) age 18 years or older, (3) first admission to the ICU, and (4) complete documentation of both dysphagia and delirium. This meant that all ischemic stroke patients enrolled in our study underwent a thorough assessment for dysphagia and delirium. In contrast, patients were excluded if they met any of the following criteria: (1) ICU stay of less than 1 day, and (2) patients with schizophrenia. To identify patients with ischemic stroke, we used International Classification of Diseases, Ninth Revision (ICD-9) codes 433, 434, 436, 437.0, and 437.1, and International Classification of Diseases, Tenth Revision (ICD-10) codes I63, I65, and I66 [21]. Similar to Shaw et al. [22], we did not exclude patients with dementia, severe stroke, or aphasia. The patient selection process is shown in Fig. 1, and a total of 1838 patients with critical ischemic stroke were included in the study. For more details on the patient selection process, please refer to Fig. 1.

### Data extraction

In this study, a structured query language was used to retrieve data from the EHR [23]. To ensure data quality and integrity, healthcare professionals preprocessed all data and information prior to analysis. The extracted information included several categories, including general patient information, comorbidities or neurological symptoms, laboratory parameters, treatment and medication details, and outcomes, as shown in Table 1. All laboratory variables and disease severity scores were extracted from the data first measured after the patient enters the ICU, the sedatives were extracted from the patient’s use within one day of ICU admission.

**Table 1:** A univariate comparison of patient characteristics with and without dysphagia

Variable	Overall (*n* = 1838)	Without dysphagia (*n* = 1594)	Dysphagia (*n* = 244)	P-value
**General characteristics**				
Age (years old)	70 (60, 81)	69 (59, 80)	75 (65, 85)	< 0.001*
Sex(%)				0.261
Male	909 (49.5)	797 (50.0)	112 (45.9)	
Female	929 (50.5)	797 (50.0)	132 (54.1)	
Race (%)				0.771
White	1262 (68.7)	1092 (68.5)	170 (69.7)	
Others^&^	576 (31.3)	502 (31.5)	74 (30.3)	
Language (%)				0.491
English	1682 (91.5)	1462 (91.7)	220 (90.2)	
Unknown	156 (8.5)	132 (8.3)	24 (9.8)	
Weight (kg)	77.0 (64.8, 91.0)	77.3 (65.0, 92.0)	74.9 (62.5, 87.1)	0.032*
Hospital LOS (days)	6.2 (3.4, 12.3)	6.0 (3.3, 11.9)	7.2 (4.6, 13.6)	0.001*
ICU LOS (hours)	59.52 (36.00, 119.46)	58.08 (35.76, 117.30)	66.96 (38.82, 129.06)	0.106
**Scores**				
GCS	14 (11, 15)	14 (12, 15)	12 (9, 14)	< 0.001*
SOFA	3 (1, 5)	3 (1, 5)	4 (2, 6)	< 0.001*
Braden score	16 (14, 18)	16 (15, 18)	15.00 (13, 16)	< 0.001*
**Comorbidities or neurological symptoms**				
Myocardial infarct (%)				0.862
Yes	313 (17.0)	270 (16.9)	43 (17.6)	
No	1525 (83.0)	1324 (83.1)	201 (82.4)	
Congestive heart failure (%)				0.172
Yes	466 (25.4)	395 (24.8)	71 (29.1)	
No	1372 (74.6)	1199 (75.2)	173 (70.9)	
Dementia (%)				< 0.001*
Yes	97 (5.3)	61 (3.8)	36 (14.8)	
No	1741 (94.7)	1533 (96.2)	208 (85.2)	
Chronic pulmonary disease (%)				0.411
Yes	367 (20.0)	313 (19.6)	54 (22.1)	
No	1471 (80.0)	1281 (80.4)	190 (77.9)	
Rheumatic disease (%)				0.959
Yes	63 (3.4)	54 (3.4)	9 (3.7)	
No	1775 (96.6)	1540 (96.6)	235 (96.3)	
Mild liver disease (%)				0.464
Yes	78 (4.2)	65 (4.1)	13 (5.3)	
No	1760 (95.8)	1529 (95.9)	231 (94.7)	
Diabetes (%)				0.863
Yes	560 (30.5)	484 (30.4)	76 (31.1)	
No	1278 (69.5)	1110 (69.6)	168 (68.9)	
Renal disease (%)				0.056
Yes	337 (18.3)	281 (17.6)	56 (23.0)	
No	1501 (81.7)	1313 (82.4)	188 (77.0)	
Malignant cancer (%)				0.087
Yes	142 (7.7)	116 (7.3)	26 (10.7)	
No	1696 (92.3)	1478 (92.7)	218 (89.3)	
Severe liver disease (%)				0.179
Yes	19 (1.0)	14 (0.9)	5 (2.0)	
No	1819 (99.0)	1580 (99.1)	239 (98.0)	
Atrial fibrillation (%)				0.119
Yes	587 (31.9)	498 (31.2)	89 (36.5)	
No	1251 (68.1)	1096 (68.8)	155 (63.5)	
Hypertension (%)				0.079
Yes	966 (52.6)	851 (53.4)	115 (47.1)	
No	872 (47.4)	743 (46.6)	129 (52.9)	
Sepsis (%)				0.008*
Yes	584 (31.8)	488 (30.6)	96 (39.3)	
No	1254 (68.2)	1106 (69.4)	148 (60.7)	
Depression (%)				0.873
Yes	228 (12.4)	199 (12.5)	29 (11.9)	
No	1610 (87.6)	1395 (87.5)	215 (88.1)	
Malnutrition (%)				0.009*
Yes	109 (5.9)	85 (5.3)	24 (9.8)	
No	1729 (94.1)	1509 (94.7)	220 (90.2)	
Dehydration (%)				0.009*
Yes	201 (10.9)	162 (10.2)	39 (16.0)	
No	1637 (89.1)	1432 (89.8)	205 (84.0)	
History of falls (%)				0.002*
Yes	480 (26.1)	396 (24.8)	84 (34.4)	
No	1358 (73.9)	1198 (75.2)	160 (65.6)	
History of stroke (%)				< 0.001*
Yes	133 (7.2)	99 (6.2)	34 (13.9)	
No	1705 (92.8)	1495 (93.8)	210 (86.1)	
Visual or hearing deficit (%)				< 0.001*
Yes	917 (49.9)	761 (47.7)	156 (63.9)	
No	921 (50.1)	833 (52.3)	88 (36.1)	
Aphasic (%)				< 0.001
Yes	284 (15.5)	215 (13.5)	69 (28.3)	
No	1554 (84.5)	1379 (86.5)	175 (71.7)	
**Laboratory parameters**				
White blood cell (K/uL)	9.6 (7.3, 12.6)	9.6 (7.3, 12.5)	9.7 (7.3, 13.8)	0.323
Potassium (mEq/L)	4.1 (3.8, 4.4)	4.1 (3.8, 4.4)	4.1 (3.8, 4.4)	0.781
Sodium (mEq/L)	139 (137, 141)	139 (137, 141)	140 (137, 142)	0.002*
Glucose (mg/dL)	123.0 (102.0, 157.0)	123.0 (101.0, 158.0)	120.0 (104.0, 152.5)	0.725
Albumin (g/dL)	3.8 (3.4, 4.1)	3.8 (3.4, 4.2)	3.6 (3.1, 4.0)	< 0.001*
**Treatment and drugs**				
Enteral nutrition (%)				< 0.001*
Yes	371 (20.2)	287 (18.0)	84 (34.4)	
No	1467 (79.8)	1307 (82.0)	160 (65.6)	
Mechanical thrombectomy (%)				0.233
Yes	244 (13.3)	218 (13.7)	26 (10.7)	
No	1594 (86.7)	1376 (86.3)	218 (89.3)	
Alteplase (%)				0.793
Yes	154 (8.4)	132 (8.3)	22 (9.0)	
No	1684 (91.6)	1462 (91.7)	222 (91.0)	
RRT (%)				>0.999
Yes	85 (4.6)	74 (4.6)	11 (4.5)	
No	1753 (95.4)	1520 (95.4)	233 (95.5)	
Sedatives^ (%)				0.024*
Yes	562 (30.6)	503 (31.6)	59 (24.2)	
No	1276 (69.4)	1091 (68.4)	185 (75.8)	
IMV (%)				0.214
Yes	367 (20.0)	326 (20.5)	41 (16.8)	
No	1471 (80.0)	1268 (79.5)	203 (83.2)	
**Outcomes**				
Delirium (%)				< 0.001*
Yes	798 (43.4)	650 (40.8)	148 (60.7)	
No	1040 (56.6)	944 (59.2)	96 (39.3)	
Pressure injury (%)				< 0.001*
Yes	208 (11.3)	158 (9.9)	50 (20.5)	
No	1630 (88.7)	1436 (90.1)	194 (79.5)	
Urinary tract infection (%)				< 0.001*
Yes	300 (16.3)	240 (15.1)	60 (24.6)	
No	1538 (83.7)	1354 (84.9)	184 (75.4)	
Aspiration pneumonia (%)				< 0.001*
Yes	139 (7.6)	100 (6.3)	39 (16.0)	
No	1699 (92.4)	1494 (93.7)	205 (84.0)	
30-day mortality (%)				< 0.001*
Alive	1587 (86.3)	1396 (87.6)	191 (78.3)	
Expired	251 (13.7)	198 (12.4)	53 (21.7)	
360-day mortality (%)				< 0.001*
Alive	1376 (74.9)	1229 (77.1)	147 (60.2)	
Expired	462 (25.1)	365 (22.9)	97 (39.8)	

Median and interquartile range (25th and 75th percentiles) were computed for continuous variables, and frequencies and percentages were computed for categorical variables

The Wilcoxon rank-sum test was used to compare group differences for continuous variables and chi-square tests were used to compare those of categorical variables

*Abbreviations*: LOS: Length of Stay; GCS: Glasgow Coma Score; SOFA: Sequential Organ Failure Assessment; RRT: Renal Replacement Therapy; IMV: Invasive Mechanical Ventilation

Note: ^&^Other mainly included Black, Hispanic, Asian, etc

^^^Sedatives mainly included benzodiazepines, propofol and dexmedetomidine

*Significant difference between patients with dementia with dysphagia and without dysphagia (*p* < 0.05)

To avoid potential bias, variables with more than 25% missing values, including height and lactate, were intentionally excluded (eFig. 2). For those variables with less than 25% missing data, multiple imputation was performed using the predictive mean matching method via the “mice” package of the R software, using the non-missing variables for training [24]. Survival time was calculated by subtracting the ICU admission time from the time of death for each patient. In addition, all patients included in the MIMIC-IV database were followed up for at least 1 year to obtain information on long-term outcomes.

### Exposure

In our study, we defined the exposure variable as the presence or absence of dysphagia (yes/no) in patients with ischemic stroke before their admission to the ICU. Specifically, we extracted bedside dysphagia screening data from the “chartevents” table of the MIMIC-IV database. Within the “chartevents” table, we identified the specific item ID “225118” which corresponds to the assessment of dysphagia (difficulty swallowing) [25]. This information is located in the category “Adm History/FHPA” (Admission History/Family History, Physical Assessment). The use of the EHR to identify patients with dysphagia has been validated by several studies, confirming its efficacy and reliability [26, 27].

As part of the ICU Admission Assessment, this assessment was performed by trained nurses upon admission of patients to the ICU. The nursing team primarily used an assessment procedure that included taking a medical history and screening for water swallowing. Detailed information about this assessment process is available at https://github.com/MIT-LCP/mimic-code/discussions/1538.

All patients admitted to the ICU who underwent dysphagia screening were included in the study cohort, regardless of their screening results. Dysphagia was methodically assessed and recorded for each individual, with a positive screening result categorized as indicating the presence of dysphagia and a negative result as indicating the absence of dysphagia. This approach allowed for a comprehensive and nuanced assessment of the prevalence of dysphagia within our cohort, including both patients who were positively diagnosed with dysphagia (positive cohort) and those who underwent screening but had no symptomatic evidence of the condition (negative cohort).

### Outcomes

In this study, the primary outcome of interest was delirium, which was assessed using the ICU Confusion Assessment Method (CAM-ICU) [28]. The CAM-ICU scale consists of four dimensions or characteristics that are assessed to diagnose delirium. These dimensions include:

1. Acute alteration or fluctuation in level of consciousness.
2. Inattention.
3. Altered level of consciousness.
4. Disorganized thinking.

A patient is considered positive for delirium if they meet characteristics 1 and 2, and either characteristic 3 or 4. In a meta-analysis, the CAM-ICU was shown to have high sensitivity (84%) and specificity (95%) [29]. In addition to using the CAM-ICU, nursing notes were reviewed to identify patients with delirium. Key words such as “delirium,” “confusion,” “agitation,” and “altered mental status” were used to identify cases of delirium [30, 31]. The nursing team continued to do delirium assessments as needed throughout the ICU stay and until the patient’s discharge.

Other outcomes assessed in the study included aspiration pneumonia, urinary tract infection, and pressure injury. Aspiration pneumonia and urinary tract infection were diagnosed using the ICD-9 and ICD-10 codes. The assessment of pressure injuries was performed by critical care nurses using the international staging definitions, which classifies injuries into stages (Stage I to IV), as well as unstageable and suspected deep tissue injuries [32].

### Covariates

The analysis included the following covariates: age, sex, race, Glasgow Coma Score (GCS), and Sequential Organ Failure Assessment (SOFA) score. In addition, when examining the association between dysphagia and delirium, we included five additional covariates: dementia, depression, sedation, history of falls (within three months) and visual or hearing deficit (assessed at ICU admission), which have been associated with delirium. Similarly, when examining the association between dysphagia and pressure injuries, we included the Braden score, a validated tool for predicting the risk of pressure injuries in patients.

### Statistical analysis

Our study followed the reporting recommendations outlined by STROBE (Strengthening the Reporting of Observational Studies in Epidemiology) [33]. First, we performed statistical analyses to describe and compare differences between groups based on exposure factors, specifically the presence or absence of dysphagia. The normality of continuous variables was assessed using the Shapiro-Wilk test, which indicated that the majority of these variables deviated from a normal distribution. Continuous variables were presented as medians and interquartile ranges, and the Wilcoxon rank-sum test was used to assess group differences. Categorical variables were expressed as numbers and percentages, and differences between groups were assessed using the chi-squared test. Logistic regression models were used to determine the association between dysphagia and outcome events, providing odds ratios (OR) and 95% confidence intervals (CI). In addition, Kaplan-Meier (KM) survival curves were estimated to depict 30-day and 360-day survival rates for individuals who developed delirium during critical illness, with differences in survival distributions assessed using the log-rank test. Subgroup analyses were then performed to examine the association between dysphagia and delirium within different subgroups. Specifically, we examined the association between dysphagia and delirium in subgroups based on sex (male/female), age (≥ 65 years/<65 years in adults), race (white/other), dementia (yes/no), enteral nutrition (yes/no), and atrial fibrillation (yes/no).

In addition, if a significant association between dysphagia and delirium during a patient’s critical illness is found in the primary analysis, we will conduct further investigation into explanatory factors and potential mechanisms. To this end, we used the “mediation” R package to perform a causal mediation analysis. This analysis included the calculation of average direct effects (ADE) and average causal mediation effects (ACME), which represent the direct and indirect effects of dysphagia on outcomes. To account for potential bias, we used bias-corrected nonparametric bootstrapping with 50,000 resamplings. Patient demographics, including sex, age, and race, were adjusted for in our analysis.

Finally, we performed sensitivity analyses to ensure the robustness of our findings. First, we performed propensity score matching (PSM) to further adjust for covariates and minimize the impact of potential confounding variables between the treatment and control groups in our observational study. Propensity scores were estimated using logistic regression models based on sex, age, race, GCS, SOFA scores, dementia, depression, sedatives, history of falls and visual or hearing deficit. We used nearest-neighbor matching with a 1:4 matching algorithm to create matched populations, and comparison between groups was assessed using the Wilcoxon rank-sum test or chi-squared test. Standardized mean differences (SMD) were used to assess covariate balance between subjects, with empirical evidence suggesting a threshold of less than 0.1 as an indication of optimal covariate balance [34]. Given the increased propensity for dysphagia and delirium in recurrent stroke patients, it was of paramount importance to limit the scope of the study to those experiencing their first stroke. By focusing on first-ever stroke, we were able to significantly reduce the potential confounding effects that previous strokes may have had on the occurrence of dysphagia and delirium. Therefore, we excluded patients with a history of stroke (*n* = 133) from the sensitivity analysis. In addition, we excluded patients with aphasia (*n* = 284) from our sensitivity analysis to reduce the impact of language dysfunction on assessment and potential confounding variables. Although we included potential confounders in our model, there may still be unmeasured factors that could influence the results. To address this, we introduced the E-value, a metric used to assess the likelihood of missing variables in the observed associations. The E-value helps to assess whether there may be unmeasured factors that could explain the observed associations [35]. A bigger E-value indicates greater robustness of the findings and suggests that the observed associations are less likely to be explained or overturned by confounding variables. We calculated the E-value using an online calculator available at (https://www.evalue-calculator.com/evalue/). R software (version 4.2.3, https://www.r-project.org/) was used for data processing. Statistical significance was determined using a threshold of *p* < 0.05.

## Results

### Baseline characteristics of the participants

A total of 1,838 patients diagnosed with ischemic stroke during critical illness were included in this observational study, of whom 244 presented with dysphagia. The median ICU length of stay of the study population was approximately 60 h, and the ICU length of stay of the dysphagia group was slightly longer than that of the non-dysphagia group, but the difference was not statistically significant (*P* = 0.106). The baseline characteristics of the patients were shown in Table 1, and the entire population was divided into groups based on exposure factors to compare differences (Table 1). The median age of all participants was approximately 70 years (interquartile range [IQR]: 60–81 years). Of the participants, 929 (50.5%) were female, mostly white (68.7%), and the vast majority native English speakers (91.5%). Ischemic stroke patients with dysphagia had a significantly higher median age of approximately 75 years compared to those without dysphagia (approximately 69 years). They also had lower body weight, lower serum albumin levels, longer hospital stays, and potentially worse health status (e.g., higher SOFA scores, lower GCS scores). All of these differences were statistically significant (*p* < 0.05). However, there were no statistically significant differences between the two groups with respect to sex and race. In terms of comorbidities, the dysphagia group had a higher incidence of sepsis, dementia, dehydration, and malnutrition compared to the non-dysphagia group. Enteral nutrition was used more frequently in the dysphagia group than in the non-dysphagia group. In terms of study outcomes, patients with ischemic stroke and dysphagia were more likely to experience adverse outcomes. Specifically, these individuals had higher 30-day (21.7% vs. 12.4%) and 360-day (39.8% vs. 22.9%) mortality rates, as well as higher rates of delirium (60.7% vs. 40.8%), aspiration pneumonia (16.0% vs. 6.3%), pressure injuries (20.5% vs. 9.9%), and urinary tract infections (24.6% vs. 15.1%) compared to those without dysphagia (all *p* < 0.05).

### Dysphagia is associated with an increased prevalence of delirium during critical illness

**Fig. 2:**
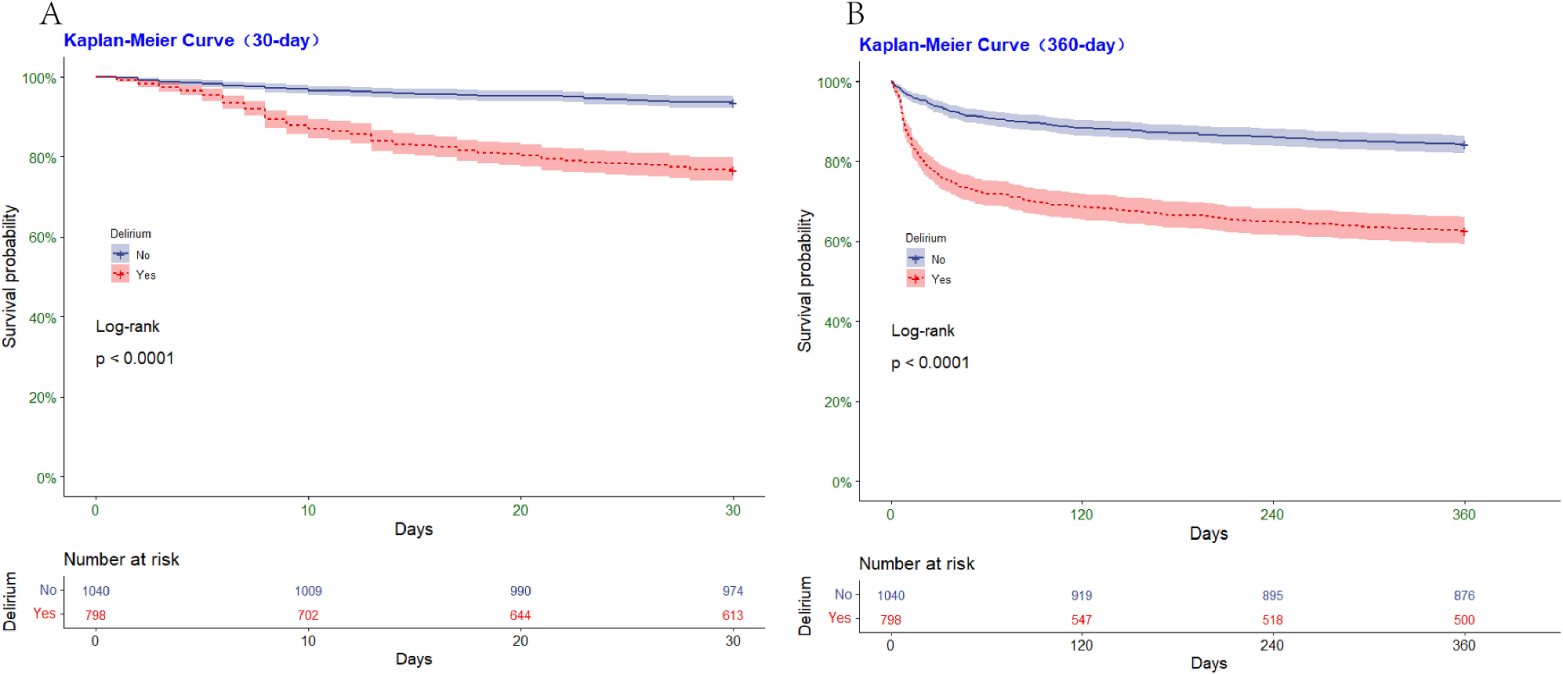
Kaplan-Meier survival curves between groups. *Note*: P-value calculated by Log-rank test ＜0.05 showed that patients with delirium have a lower survival probability than those delirium. (**A**) Represented the survival probability in 30-day; (**B**) Represented the survival probability in 360-day

Table 2 showed the association between dysphagia and the risk of adverse outcomes during critical illness. After adjusting for confounding factors, dysphagia was found to be significantly associated with an increased prevalence of delirium during critical illness (adjusted odds ratio [aOR]: 1.48; 95% CI: 1.07–2.05; *p* = 0.018; E-value = 1.73) (Table 2). Furthermore, multifactorial binary logistic regression analysis showed that dysphagia was associated with a higher prevalence of aspiration pneumonia (aOR: 2.39; 95% CI: 1.56–3.61; *p* < 0.001; E-value = 4.21) and pressure injury (aOR: 1.69; 95% CI: 1.13–2.50; *p* = 0.009; E-value = 2.77). However, after adjusting for confounders, there was no statistically significant association between dysphagia and urinary tract infection (aOR = 1.37, 95% CI = 0.97–1.91, *p* = 0.071). Kaplan-Meier survival curves (Fig. 2) showed differences in the probability of survival between patients with and without delirium during critical illness (log-rank test; *p* < 0.001). Both at 30 days (Fig. 2A) and 360 days (Fig. 2B), the probability of survival was significantly lower in the delirium group compared to the non-delirium group (*p* < 0.001).

**Table 2:** Logistic regression: association between dysphagia and primary/secondary study outcomes

	Without dysphagia	Dysphagia	P-value	E-value (lower limit of the 95% CIs)
		ORs (95% CIs)		
Delirium^●^				
Unadjusted Adjusted	Reference Reference	2.24 (1.70, 2.96) 1.48 (1.07, 2.05)	< 0.001* 0.018*	Not applicable 1.73 (1.22)
Aspiration pneumonia^&^				
Unadjusted Adjusted	Reference Reference	2.84 (1.89, 4.20) 2.39 (1.56, 3.61)	< 0.001* < 0.001*	Not applicable 4.21 (2.49)
Urinary tract infection^&^				
Unadjusted Adjusted	Reference Reference	1.71 (1.28, 2.29) 1.37 (0.97, 1.91)	< 0.001* 0.071	Not applicable Not applicable
Pressure injury^$^				
Unadjusted Adjusted	Reference Reference	2.34 (1.64, 3.31) 1.69 (1.13, 2.50)	< 0.001* 0.009*	Not applicable 2.77 (1.51)

*Abbreviations*: ORs: Odds Ratios; CIs: Confidence Intervals

Note: Logistic regression models were used to calculate odds ratios (ORs) with 95% confidence intervals (CIs)

*Significant difference between patients with dysphagia and without dysphagia (*p* < 0.05)

^●^Delirium was adjusted for age, sex, race, dementia, depression, sedatives, history of falls, visual or hearing deficit, SOFA and GCS

^&^Aspiration pneumonia and urinary tract infection were adjusted for age, sex, race, GCS and SOFA

^$^Pressure Injury was adjusted for age, sex, race, GCS, SOFA and Braden score

### Causal mediation analysis

**Fig. 3:**
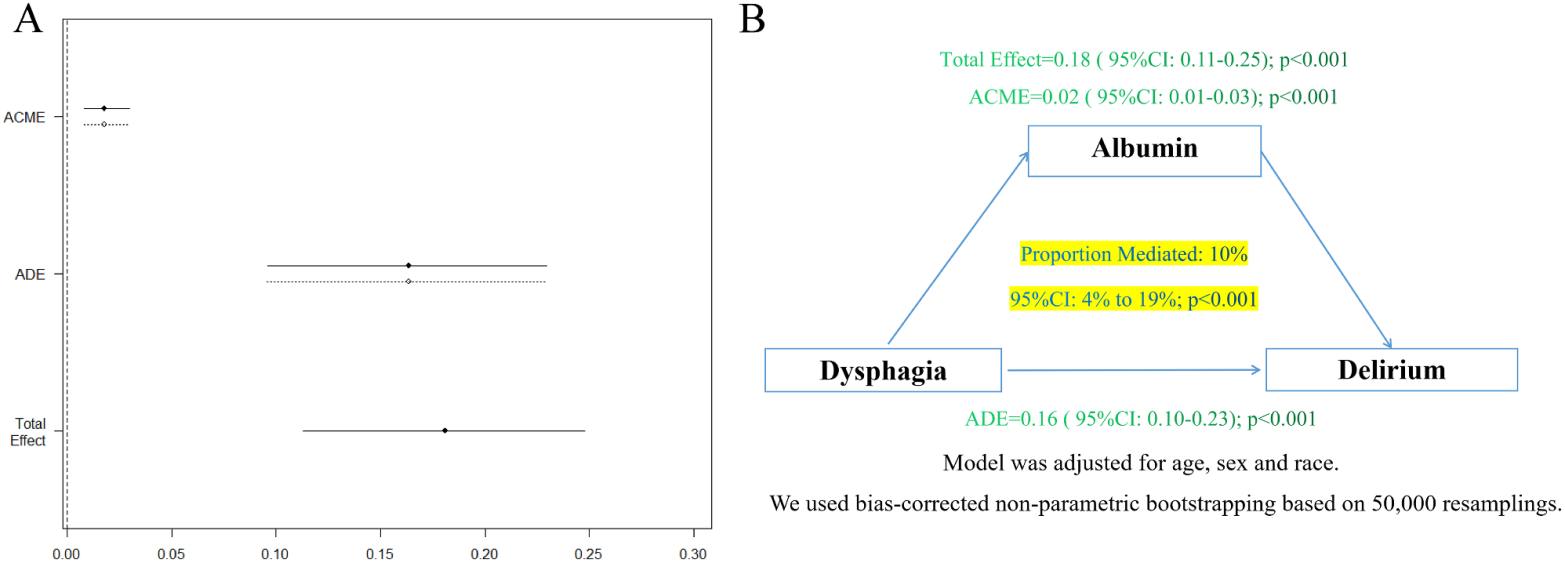
Causal mediation analysis. (**A**) Process diagram for mediation analysis. (**B**) directed acyclic graph. Note: Diagram showing components of the mediation model testing the effect of dysphagia lead on delirium mediated by serum albumin level

To further examine the association between dysphagia and adverse outcomes, particularly delirium, we conducted a causal mediation analysis using serum albumin levels measured during the patient’s initial admission to the ICU as a mediating factor (Fig. 3A). After adjusting for demographic variables such as sex, age, and race (Fig. 3B), we found that serum albumin levels partially mediated the association between dysphagia and delirium in critically ill patients with ischemic stroke (ACME: 0.02, 95% CI: 0.01 to 0.03; *p* < 0.001) (Fig. 3). The association between increased risk of dysphagia and delirium was found to be partially mediated by serum albumin, accounting for approximately 10% (95%CI: 4-19%, *p* < 0.001) of the mediation. A similar pattern was observed in the analysis of aspiration pneumonia and pressure injury (eFig. 3). In addition, mediation analysis showed that aspiration pneumonia may be associated with dysphagia and delirium (eFig. 4).

### Subgroup analysis

**Fig. 4:**
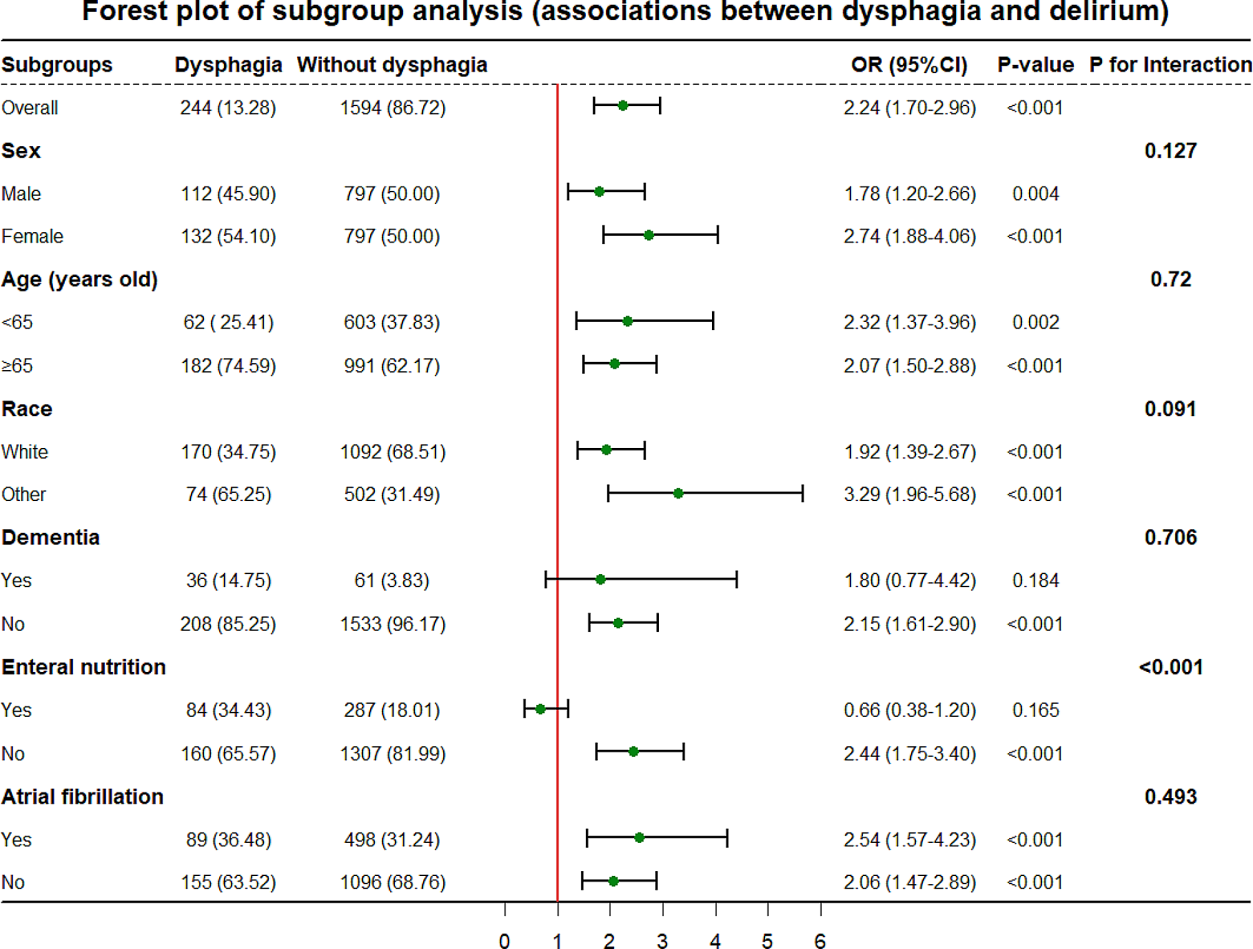
Forest plot for subgroup analysis. Note: Represented the association between dysphagia and delirium among subpopulations. Abbreviations: OR: Odds Ratio; CI: Confidence Interval

A subgroup analysis was performed in this study to examine the association between dysphagia and the primary outcome (delirium) within a specific population (Fig. 4). The results showed that when examining the association between dysphagia and delirium in patients with ischemic stroke, no statistically significant association was observed in the subpopulation with dementia and the subpopulation receiving enteral nutrition (both *p* > 0.05). In addition, a significant interaction was observed between dysphagia and enteral nutrition (*p* < 0.001).

### Sensitivity analysis

The baseline data of the two groups after PSM are shown in (eTable 1). The SMD after PSM for covariates showed a better balance between the groups than that of the original cohort (eFig. 5). By performing univariate and multivariate logistic regression on the matched population, consistent results were obtained with the original population (eTable 2), demonstrating that dysphagia was associated with adverse outcomes during critical illness in patients with ischemic stroke (all *p* < 0.05). These results demonstrate the robustness of our findings. We also found consistent results when we analyzed the association between dysphagia and delirium after excluding patients with previous stroke and those with aphasia (eTable 3).

In our study, after adjusting for measured confounders, we found that dysphagia was associated with an increased risk of delirium, with an OR of 1.48 (95% CI: 1.07–2.05). To assess the robustness of this finding to potential unmeasured or residual confounding, we calculated the E-value. The E-value for our observed association was 1.73, with the lower limit of the 95% CI at 1.22. Following the method of VanderWeele et al. [35], this means that the observed OR of 1.48 could be explained away by an unmeasured confounder that was associated with both the exposure and the outcome by a OR of 1.73-fold each, above and beyond the measured confounders, to fully account for the observed association. In other words, relatively strong confounding would be required to fully explain the observed exposure-outcome association of OR = 1.48.

## Discussion

### Main findings

Despite the high prevalence of dysphagia in patients with ischemic stroke during critical illness, there are few studies that comprehensively examine the association between dysphagia and patient characteristics and clinical outcomes in this specific population. To the best of our knowledge, our study is the first population-based analysis to examine the mediated effects and association between dysphagia and delirium in critically ill ischemic stroke patients using a large critical care database in the United States. Our findings provide evidence that dysphagia is an independent risk factor for delirium after critical illness and also increases the likelihood of developing aspiration pneumonia and pressure injuries. In addition, our causal mediation analysis shows that serum albumin levels partially mediate the association between dysphagia and delirium in critically ill patients with ischemic stroke.

### Comparison with other studies, interpretation of results and clinical implications

Dysphagia, a common and serious problem among adults, has a profound impact on their nutritional well-being, as well as the potential for respiratory infections, pneumonia and related complications [36]. Previous studies have shown that dysphagia may be an independent risk factor for the development of delirium [12, 26, 37, 38], and we have reached similar conclusions in patients with ischemic stroke in the ICU. It may be caused by several interacting factors. First, dysphagia may induce gastrointestinal dysfunction, thereby influencing the brain-gut axis. Disruption of this axis may induce inflammatory responses, disrupt neurotransmitter balance, and contribute to psychological deterioration, ultimately increasing susceptibility to delirium [39]. Second, dysphagia can precipitate aspiration and subsequent pulmonary infection, thereby initiating a systemic inflammatory response [40]. Inflammatory markers such as interleukin-1B (IL-1B), interleukin-6 (IL-6), and tumor necrosis factor-a (TNF-a) have been observed to be elevated in patients with delirium [41]. Inflammation may adversely affect the central nervous system, thereby precipitating the onset of delirium. Third, dysphagia can lead to malnutrition and metabolic disturbances, including hypoglycemia and dehydration [17]. These disturbances adversely affect the cerebral energy supply, thereby increasing the likelihood of delirium. Finally, dysphagia may result from inadequate management of delirium during hospitalization, lack of awareness of delirium, or the use of antipsychotic medications, all of which may contribute to the occurrence of dysphagia [42]. In addition, the KM survival analysis showed a significant association between delirium and increased 30-day and 360-day mortality, further supporting the findings of Klimiec-Moskal et al. and underscoring the detrimental impact of delirium on the prognosis of ischemic stroke patients [43].

In our study, we also made a significant discovery regarding the partial mediation of serum albumin levels in the association between dysphagia and delirium (ACME: 0.02, 95% CI: 0.01 to 0.03; *p* < 0.001). Specifically, dysphagia in stroke patients may lead to challenges in adequate intake of essential nutrients, resulting in malnutrition. This in turn leads to a decrease in serum albumin levels. The presence of malnutrition can further deteriorate the patient’s physiological state and negatively impact neurological function, thereby increasing the risk of delirium [44]. Wang et al. demonstrated that early implementation of protein supplementation effectively reduced mortality in critically ill stroke patients, underscoring the importance of ensuring adequate nutritional availability for the management and recovery of acutely ill patients [45]. These findings provide preliminary evidence that contributes to a better understanding of how dysphagia influences the onset and progression of delirium. By increasing nutritional intake and maintaining normal albumin levels in patients with dysphagia, we may be able to improve immune function, preserve neurological integrity, and facilitate tissue repair and regeneration, thereby potentially reducing the incidence of delirium.

As previously documented, patients with dysphagia are at increased risk for both aspiration pneumonia and pressure injuries [17, 46, 47]. Our study showed similar results, establishing dysphagia as a potential independent risk factor for these conditions. In patients with ischemic stroke in the ICU, the ischemic stroke itself may reduce the cough reflex and impair the ability to clear airway secretions [48]. This, combined with dysphagia, increases susceptibility to aspiration pneumonia. In addition, patients with dysphagia may experience inadequate nutritional intake, muscle weakness and muscle wasting, making them more susceptible to skin and soft tissue damage and contributing to the development of pressure injuries [49]. To reduce the incidence of aspiration pneumonia and pressure injuries and improve patient prognosis, it is critical to perform early assessment of swallowing function in ischemic stroke patients in the ICU, promote multidisciplinary collaboration for swallowing intervention and rehabilitation, monitor patients closely, and intervene promptly when necessary.

A previous study by Bond et al. found a significant association between dysphagia and urinary tract infection [50]. However, our study did not identify dysphagia as an independent risk factor for UTI. We note that urinary tract infection is a multifaceted problem influenced by various factors such as catheterization, sex, age, chronic disease, and immunosuppression. Although dysphagia may contribute to an increased risk of UTI, it is unlikely to be a sole independent risk factor in patients with ischemic stroke in the ICU [51]. Although our study did not find an independent association between dysphagia and UTI in critically ill stroke patients, this does not mean that dysphagia has no impact on overall systemic health. Dysphagia remains a significant problem that can lead to other complications and affect patient recovery and quality of life. Therefore, it is imperative that dysphagia in critically ill stroke patients be given sufficient attention and that appropriate interventions be implemented.

We performed a subgroup analysis and found that the association between dysphagia and delirium did not differ significantly between patients with dementia and those receiving enteral nutrition. We speculate that the lack of statistical significance in the dementia subgroup may be due to the small sample size (dysphagia group *n* = 36, no dysphagia group *n* = 61), which may have limited the statistical power to detect significant differences. Future studies with larger sample sizes are needed to more conclusively explore the relationship between dysphagia and delirium in this specific population. In addition, patients with dysphagia who receive enteral nutrition may experience some degree of nutritional and metabolic improvement, which may reduce the risk of delirium. These factors may partially offset the effect of dysphagia on delirium risk [52]. Therefore, future research should pay special attention to the potential influence of enteral nutrition in the development of delirium and investigate the mechanism by which enteral nutrition works. Such efforts would facilitate the formulation of more precise and personalized strategies for nutritional intervention.

There is a lack of effective clinical assessment and management approaches for dysphagia and its associated symptoms in ischemic stroke patients [53]. While studies have used screening tools such as the Functional Oral Intake Scale, Swallowing Ability and Function Evaluation, bedside swallowing assessment, imaging swallowing assessment, and swallow muscle electrogram, these methods have limitations in assessing swallowing function and abnormalities in patients with dysphagia [54–56]. For example, the Horizontal Swallowing Assessment and Swallowing Capacity Assessment scales focus primarily on swallowing ability and feeding status, neglecting aspects such as swallowing coordination, muscle movement, and food passage through the esophagus. Although imaging swallowing assessments, and swallowing muscle electrograms provide more detailed information, they require additional time and expertise and are not commonly used in clinical practice [12]. Therefore, a comprehensive understanding of a patient’s swallowing problems and the formulation of personalized management strategies can be achieved by combining assessment tools and methods with clinical observation and professional judgment of the healthcare team. Furthermore, the implementation of appropriate dietary regimens that include texture modification, food consistency adjustments, and the use of supplemental feeding devices is a common nutritional management strategy for patients with dysphagia [57]. These strategies aim to ensure safe ingestion and adequate nutrient intake to prevent malnutrition and weight loss. However, several systematic reviews and meta-analyses indicate insufficient evidence to support the substantial impact of food texture modification and fluid concentration adjustment on improving swallowing function recovery, reducing aspiration errors, and mitigating complications such as respiratory infections [58–60]. Therefore, further research and validation of nutritional management strategies for patients with dysphagia is warranted to determine the most effective interventions. In particular, patients with dysphagia in critical care settings present unique challenges due to their unique condition and complex nutritional needs, making this an important area for future research.

Given the clinical significance of delirium and dysphagia in patients with ischemic stroke, it is strongly recommended that assessment of dysphagia be incorporated into the routine nursing assessment and physical examination of these patients in the ICU. This approach would allow for early identification and intervention of dysphagia, implementation of targeted nutritional support interventions, and ultimately improve clinical outcomes and prognosis in individuals with ischemic stroke. Early recognition and treatment of dysphagia may prevent the onset of malnutrition and allow for the implementation of appropriate interventions to maintain the patient’s nutritional status. In addition, early recognition of dysphagia can help reduce the risk of complications such as pressure ulcers and pneumonia. Therefore, integrating the assessment of dysphagia into early care practices is a critical measure to improve the quality of patient care and promote recovery in individuals with ischemic stroke.

### Strengths and limitations

This study has some notable strengths. First, we performed multiple sensitivity analyses to ensure the robustness of our findings. Second, subgroup analyses were performed to examine variations among different subpopulations, providing a deeper understanding of the association between dysphagia and delirium. This knowledge may help clinical staff to identify high-risk or special populations. Third, we used a causal mediator approach to evaluate potential explanatory factors and underlying mechanisms, thereby providing guidance to clinical health care providers and managers in developing intervention strategies. Finally, we used the CAM-ICU in combination with nursing note reviews to more accurately identify delirium. We also implemented a standardized dysphagia screening for all patients, which improved the accuracy of our dysphagia assessment. These methods improved the identification of high-risk patients and supported personalized care in the ICU.

Despite the aforementioned strengths, our study has several limitations. First, it should be noted that this study was conducted as a single-center retrospective cohort study, specifically among critically ill patients admitted to a tertiary academic center in the United States. Therefore, there is a possibility of underrepresentation and external data are needed to validate our findings. Caution should be exercised when extrapolating the results to other populations. Second, the information used in this study was collected from the EHR system. Due to limitations in the availability of database information, certain important covariates, such as the National Institutes of Health Stroke Scale and body mass index, were inevitably missing from our analysis. Third, our study focused only on patients who developed delirium during their ICU stay, potentially missing individuals who presented with delirium on admission. This may have resulted in missing information or introduced bias into our analysis. Fourth, despite our use of variables potentially indicative of neurological function for subsequent analysis (e.g., history of falls, visual/hearing deficit) within the scope of the study, the issue of stroke severity was not conclusively addressed. Further compounding this was the absence of a specific ischemic stroke diagnosis time within the database. As a result, the temporal interval between stroke onset and ICU admission remained undefined, potentially compromising the validity and interpretability of the study results. Fifth, relying solely on albumin as an indicator in a mediation analysis may not adequately capture the nutritional status of the patient, as albumin represents only one facet of the overall nutritional picture. In addition, albumin levels can be affected by a variety of factors, such as infection and underlying disease, potentially confounding the relationship between albumin and delirium. To establish a valid causal mediation, the diagnosis of aspiration pneumonia should ideally occur after dysphagia and before the diagnosis of delirium. However, the precise diagnostic timeline for aspiration pneumonia remains unspecified in the database, presenting only an association and limiting the scope for causal inference. Finally, it is important to recognize that swallowing in stroke patients is a multifaceted issue. Simply categorizing subjects into two groups (dysphagia and no dysphagia) within our model may differ from the complexity of the real clinical setting.

Given these limitations, future studies could focus on conducting multicenter, prospective cohort studies to improve the representativeness of our findings and allow for more robust data collection. These studies could use a more comprehensive set of parameters and measures of stroke severity, allowing for a more nuanced understanding of the relationship between dysphagia and delirium. Furthermore, exploring the complexity of dysphagia in stroke patients beyond a binary categorization may provide more clinically relevant insights into the effective management of these patients.

## Conclusions

In conclusion, our study successfully demonstrated a significant association between dysphagia in ischemic stroke patients and a higher prevalence of critical illness delirium. Furthermore, ischemic stroke patients with dysphagia showed an increased incidence of aspiration pneumonia and pressure injury compared to ischemic stroke patients without dysphagia. Given the negative impact of dysphagia in patients with ischemic stroke on ICU admission, we strongly recommend that assessment of dysphagia be included in the standard admission assessment for these individuals.

### Electronic supplementary material

Below is the link to the electronic supplementary material.

Supplementary Material 1: Supplementary tables and figures

## Data Availability

The data were available on the MIMIC-IV website at https://mimic.physionet.org/. The data in this article can be reasonably applied to the corresponding author.

## Abbreviations

ACME: Average Causal Mediation Effects
ADE: Average Direct Effects
aOR: adjusted Odds Ratios
CAM-ICU: ICU Confusion Assessment Method
CI: Confidence Intervals
EHR: Electronic Health Records
GCS: Glasgow Coma Score
ICD: International Classification of Diseases
ICU: Intensive Care Units
KM: Kaplan-Meier
MIMIC-IV: Medical Information Mart for Intensive Care IV
OR: Odds Ratios
PSM: Propensity Score Matching
SOFA: Sequential Organ Failure Assessment
STROBE: Strengthening the Reporting of Observational Studies in Epidemiology
