# Supplementary material for: Does ICU admission dysphagia independently contribute to delirium risk in ischemic stroke patients? Results from a cohort study
Source: BMC Psychiatry. 2024 Jan 23;24:65. doi: 10.1186/s12888-024-05520-w (PMC10804594; doi:10.1186/s12888-024-05520-w)
Supplement: Supplementary file 1 — Supplementary Material 1: Supplementary tables and figures [file 12888_2024_5520_MOESM1_ESM.docx]

**eTable 1** A univariate comparison of patient characteristics after propensity score matching with and without dysphagia.

|  | Overall (n=1220) | Without dysphagia (n=976) | Dysphagia (n=244) | P-value |
| --- | --- | --- | --- | --- |
| **General characteristics** | | | | |
| Age (years old) | 73.95 (63.45, 83.76) | 73.61 (62.96, 83.63) | 75.27 (64.95, 84.58) | 0.097 |
| Sex(%) |  |  |  | 0.576 |
| Male | 638 (52.3) | 506 (51.8) | 132 (54.1) |  |
| Female | 582 (47.7) | 470 (48.2) | 112 (45.9) |  |
| Race (%) |  |  |  | >0.999 |
| White | 852 (69.8) | 682 (69.9) | 170 (69.7) |  |
| Others^&^ | 368 (30.2) | 294 (30.1) | 74 (30.3) |  |
| Language (%) |  |  |  | 0.632 |
| English | 1112 (91.1) | 892 (91.4) | 220 (90.2) |  |
| Unknown | 108 (8.9) | 84 (8.6) | 24 (9.8) |  |
| Weight (kg) | 75.67 (63.60, 89.90) | 75.85 (63.65, 90.00) | 75.00 (62.45, 87.33) | 0.288 |
| Hospital LOS (days) | 7.02 (3.90, 13.61) | 6.95 (3.79, 13.60) | 7.19 (4.57, 13.62) | 0.198 |
| ICU LOS (hours) | 68.88 (40.32, 141.12) | 69.60 (40.56, 145.92) | 66.96 (38.88, 129.12) | 0.598 |
| **Scores** | | | | |
| GCS | 13.00 (9.00, 14.00) | 14.00 (10.00, 14.00) | 12.00 (9.00, 14.00) | <0.001 |
| SOFA | 3.00 (2.00, 5.00) | 3.00 (2.00, 5.00) | 4.00 (2.00, 6.00) | 0.003 |
| Braden score | 16.00 (14.00, 18.00) | 16.00 (14.00, 18.00) | 15.00 (13.00, 16.00) | <0.001 |
| **Comorbidities or neurological symptoms** | | | | |
| Myocardial infarct (%) |  |  |  | 0.7 |
| Yes | 228 (18.7) | 185 (19.0) | 43 (17.6) |  |
| No | 992 (81.3) | 791 (81.0) | 201 (82.4) |  |
| Congestive heart failure (%) |  |  |  | 0.962 |
| Yes | 354 (29.0) | 283 (29.0) | 71 (29.1) |  |
| No | 866 (71.0) | 693 (71.0) | 173 (70.9) |  |
| Dementia (%) |  |  |  | <0.001 |
| Yes | 97 (8.0) | 61 (6.2) | 36 (14.8) |  |
| No | 1123 (92.0) | 915 (93.8) | 208 (85.2) |  |
| Chronic pulmonary disease (%) |  |  |  | 0.421 |
| Yes | 245 (20.1) | 191 (19.6) | 54 (22.1) |  |
| No | 975 (79.9) | 785 (80.4) | 190 (77.9) |  |
| Rheumatic disease (%) |  |  |  | >0.999 |
| Yes | 43 (3.5) | 34 (3.5) | 9 (3.7) |  |
| No | 1177 (96.5) | 942 (96.5) | 235 (96.3) |  |
| Mild liver disease (%) |  |  |  | 0.657 |
| Yes | 56 (4.6) | 43 (4.4) | 13 (5.3) |  |
| No | 1164 (95.4) | 933 (95.6) | 231 (94.7) |  |
| Diabetes (%) |  |  |  | 0.702 |
| Yes | 395 (32.4) | 319 (32.7) | 76 (31.1) |  |
| No | 825 (67.6) | 657 (67.3) | 168 (68.9) |  |
| Renal disease (%) |  |  |  | 0.311 |
| Yes | 249 (20.4) | 193 (19.8) | 56 (23.0) |  |
| No | 971 (79.6) | 783 (80.2) | 188 (77.0) |  |
| Malignant cancer (%) |  |  |  | 0.063 |
| Yes | 93 (7.6) | 67 (6.9) | 26 (10.7) |  |
| No | 1127 (92.4) | 909 (93.1) | 218 (89.3) |  |
| Severe liver disease (%) |  |  |  | 0.413 |
| Yes | 16 (1.3) | 11 (1.1) | 5 (2.0) |  |
| No | 1204 (98.7) | 965 (98.9) | 239 (98.0) |  |
| Atrial fibrillation (%) |  |  |  | 0.964 |
| Yes | 449 (36.8) | 360 (36.9) | 89 (36.5) |  |
| No | 771 (63.2) | 616 (63.1) | 155 (63.5) |  |
| Hypertension (%) |  |  |  | 0.069 |
| Yes | 641 (52.5) | 526 (53.9) | 115 (47.1) |  |
| No | 579 (47.5) | 450 (46.1) | 129 (52.9) |  |
| Sepsis (%) |  |  |  | 0.525 |
| Yes | 456 (37.4) | 360 (36.9) | 96 (39.3) |  |
| No | 764 (62.6) | 616 (63.1) | 148 (60.7) |  |
| Depression (%) |  |  |  | 0.628 |
| Yes | 132 (10.8) | 103 (10.6) | 29 (11.9) |  |
| No | 1088 (89.2) | 873 (89.4) | 215 (88.1) |  |
| Malnutrition (%) |  |  |  | 0.05 |
| Yes | 83 (6.8) | 59 (6.0) | 24 (9.8) |  |
| No | 1137 (93.2) | 917 (94.0) | 220 (90.2) |  |
| Dehydration (%) |  |  |  | 0.088 |
| Yes | 153 (12.5) | 114 (11.7) | 39 (16.0) |  |
| No | 1067 (87.5) | 862 (88.3) | 205 (84.0) |  |
| History of falls (%) |  |  |  | 0.61 |
| Yes | 381 (31.2) | 301 (30.8) | 80 (32.8) |  |
| No | 839 (68.8) | 675 (69.2) | 164 (67.2) |  |
| History of stroke (%) |  |  |  | 0.002 |
| Yes | 106 (8.7) | 72 (7.4) | 34 (13.9) |  |
| No | 1114 (91.3) | 904 (92.6) | 210 (86.1) |  |
| Visual or hearing deficit (%) |  |  |  | 0.472 |
| Yes | 743 (60.9) | 589 (60.3) | 90 (63.1) |  |
| No | 477/ (39.1) | 387 (39.7) | 154 (36.9) |  |
| Aphasic (%) |  |  |  | <0.001 |
| Yes | 236 (19.3) | 167 (17.1) | 69 (28.3) |  |
| No | 984 (80.7) | 809 (82.9) | 175 (71.7) |  |
| **Laboratory parameters** | | | | |
| White blood cell (K/uL) | 9.60 (7.40, 12.90) | 9.60 (7.40, 12.70) | 9.70 (7.30, 13.80) | 0.582 |
| Red blood cell (m/uL) | 3.94 (3.40, 4.47) | 3.95 (3.40, 4.47) | 3.91 (3.44, 4.50) | 0.962 |
| Platelet (K/uL) | 214.00 (166.00, 265.00) | 214.00 (166.00, 264.25) | 219.00 (163.50, 270.25) | 0.518 |
| Hemoglobin (g/dL) | 11.90 (10.10, 13.40) | 11.90 (10.20, 13.40) | 11.90 (10.10, 13.40) | 0.736 |
| Potassium (mEq/L) | 4.10 (3.80, 4.50) | 4.10 (3.80, 4.50) | 4.10 (3.80, 4.43) | 0.776 |
| Sodium (mEq/L) | 139.00 (137.00, 142.00) | 139.00 (136.00, 141.00) | 140.00 (137.00, 142.00) | 0.001 |
| Glucose (mg/dL) | 125.00 (103.00, 160.00) | 127.00 (103.00, 161.00) | 120.00 (104.00, 152.50) | 0.213 |
| Albumin (g/dL) | 3.60 (3.30, 4.00) | 3.60 (3.30, 4.00) | 3.50 (3.18, 4.00) | 0.034 |
| **Treatment and drugs** | | | | |
| Enteral nutrition (%) |  |  |  | 0.001 |
| Yes | 313 (25.7) | 229 (23.5) | 84 (34.4) |  |
| No | 907 (74.3) | 747 (76.5) | 160 (65.6) |  |
| Mechanical thrombectomy (%) |  |  |  | 0.552 |
| Yes | 146 (12.0) | 120 (12.3) | 26 (10.7) |  |
| No | 1074 (88.0) | 856 (87.7) | 218 (89.3) |  |
| Alteplase (%) |  |  |  | 0.718 |
| Yes | 120 (9.8) | 98 (10.0) | 22 (9.0) |  |
| No | 1100 (90.2) | 878 (90.0) | 222 (91.0) |  |
| RRT (%) |  |  |  | 0.591 |
| Yes | 66 (5.4) | 55 (5.6) | 11 (4.5) |  |
| No | 1154 (94.6) | 921 (94.4) | 233 (95.5) |  |
| Sedatives^ (%) |  |  |  | 0.987 |
| Yes | 298 (24.4) | 239 (24.5) | 59 (24.2) |  |
| No | 922 (75.6) | 737 (75.5) | 185 (75.8) |  |
| IMV (%) |  |  |  | 0.043 |
| Yes | 266 (21.8) | 225 (23.1) | 41 (16.8) |  |
| No | 954 (78.2) | 751 (76.9) | 203 (83.2) |  |
| **Outcomes** | | | | |
| Delirium (%) |  |  |  | 0.001 |
| Yes | 623 (51.1) | 475 (48.7) | 148 (60.7) |  |
| No | 597 (48.9) | 501 (51.3) | 96 (39.3) |  |
| Pressure injury (%) |  |  |  | 0.004 |
| Yes | 176 (14.4) | 126 (12.9) | 50 (20.5) |  |
| No | 1044 (85.6) | 850 (87.1) | 194 (79.5) |  |
| Urinary tract infection (%) |  |  |  | 0.019 |
| Yes | 233 (19.1) | 173 (17.7) | 60 (24.6) |  |
| No | 987 (80.9) | 803 (82.3) | 184 (75.4) |  |
| Aspiration pneumonia (%) |  |  |  | <0.001 |
| Yes | 119 (9.8) | 80 (8.2) | 39 (16.0) |  |
| No | 1101 (90.2) | 896 (91.8) | 205 (84.0) |  |
| 30-day mortality (%) |  |  |  | 0.02 |
| Alive | 1018 (83.4) | 827 (84.7) | 191 (78.3) |  |
| Expired | 202 (16.6) | 149 (15.3) | 53 (21.7) |  |
| 360-day mortality (%) |  |  |  | <0.001 |
| Alive | 852 (69.8) | 705 (72.2) | 147 (60.2) |  |
| Expired | 368 (30.2) | 271 (27.8) | 97 (39.8) |  |

Abbreviations: LOS: Length of Stay; GCS: Glasgow Coma Score; SOFA: Sequential Organ Failure Assessment; RRT: Renal Replacement Therapy; IMV: Invasive Mechanical Ventilation.

Note:

^&^Other mainly included Black, Hispanic, Asian, etc;

^Sedatives mainly included benzodiazepines, propofol and dexmedetomidine;

Median and interquartile range (25th and 75th percentiles) were computed for continuous variables, and frequencies and percentages were computed for categorical variables.

The Wilcoxon rank-sum test was used to compare group differences for continuous variables and chi-square tests were used to compare those of categorical variables.

**eTable 2** Logistic regression after propensity score matching: association between dysphagia and primary/secondary study outcomes.

|  | No-dysphagia | Dysphagia | P-value |
| --- | --- | --- | --- |
|  |  | ORs (95% CIs) |  |
| Delirium^●^  PSM model  Adjusted PSM model | Reference  Reference | 1.62 (1.22, 2.17)  1.41 (1.02, 1.96) | <0.001*  0.039* |
| Aspiration pneumonia^&^  PSM model  Adjusted PSM model | Reference  Reference | 2.13 (1.40, 3.20)  2.13 (1.39, 3.24) | <0.001*  <0.001* |
| Pressure injury^$^  PSM model  Adjusted PSM model | Reference  Reference | 1.74 (1.20, 2.49)  1.52 (1.01, 2.27) | 0.003*  0.040* |

Abbreviations: ORs: Odds Ratios; CIs: Confidence Intervals; PSM: Propensity Score Matching.

Note:

Logistic regression models were used to calculate odds ratios (ORs) with 95% confidence intervals (CIs).

*Significant difference between patients with dysphagia and without dysphagia (p < 0.05).

^●^Delirium was adjusted for age, sex, race, dementia, depression, sedatives, history of falls, visual or hearing deficit, SOFA and GCS;

^&^Aspiration pneumonia was adjusted for age, sex, race, GCS and SOFA;

^$^Pressure Injury was adjusted for age, sex, race, GCS, SOFA and Braden scale.

**eTable 3** Sensitivity analysis: Association between dysphagia and delirium after exclusion of specific populations.

|  | Without dysphagia | Dysphagia | P-value |
| --- | --- | --- | --- |
|  |  | ORs (95% CIs) |  |
| Delirium^●^  Model 1  Unadjusted  Adjusted | Reference  Reference | 2.19 (1.64, 2.95)  1.44 (1.02, 2.03) | <0.001*  0.040* |
| Delirium^●^  Model 2  Unadjusted  Adjusted | Reference  Reference | 2.47 (1.80, 3.41)  1.79 (1.24, 2.59) | <0.001*  0.002* |

Abbreviations: ORs: odds ratios; CIs: confidence intervals.

Note:

In model 1, patients with a history of stroke were excluded (n = 133), and in model 2, patients with aphasia were excluded (n = 284).

Logistic regression models were used to calculate odds ratios (ORs) with 95% confidence intervals (CIs).

*Significant difference between patients with dysphagia and without dysphagia (p < 0.05).

^●^Adjusted for age, sex, race, dementia, depression, sedatives, history of falls, visual or hearing deficit, SOFA and GCS;

**eFigure.1 A certificate to access the MIMIC-IV database.**


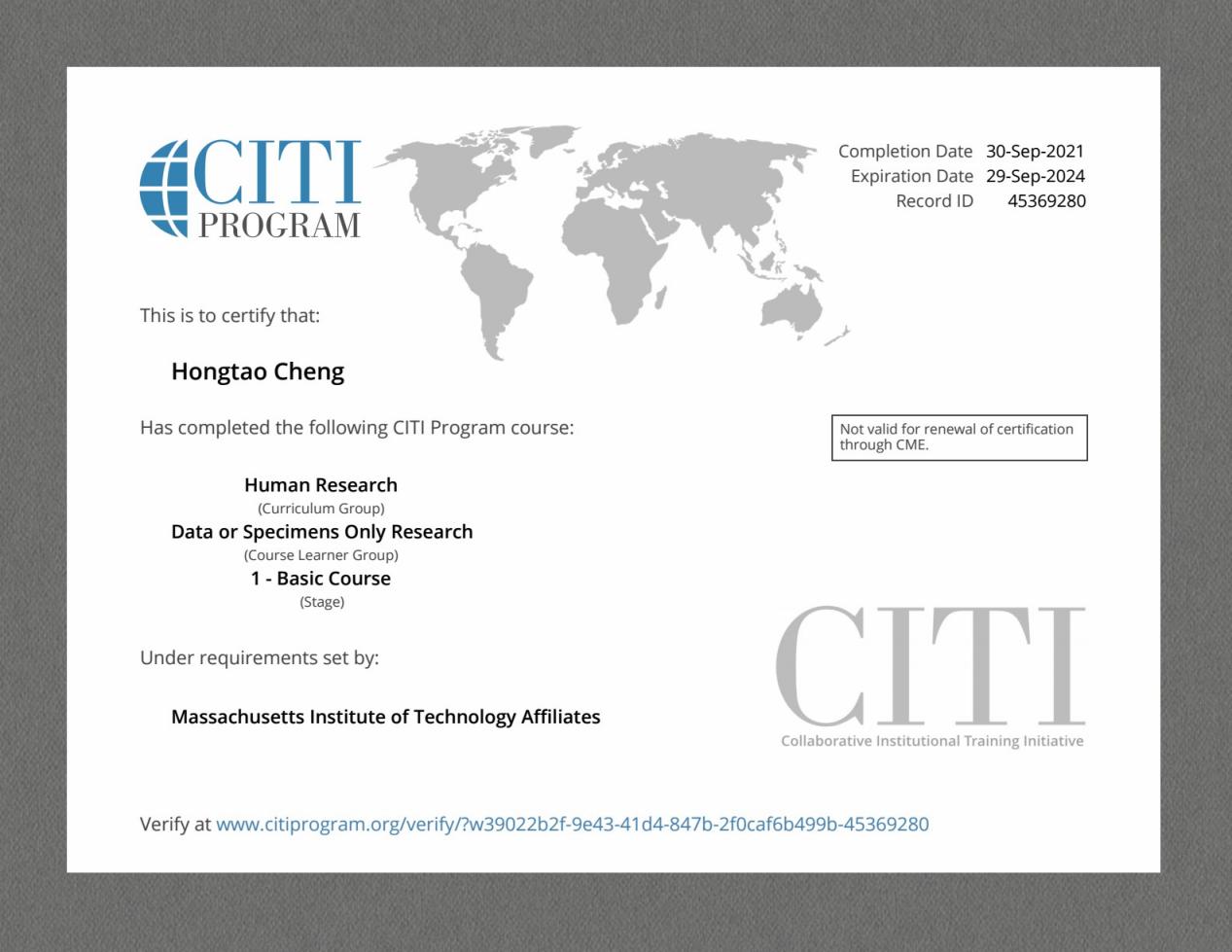


Note: One of our authors (Hongtao Cheng) received a certificate to access the database after completing the training (record ID: 45369280).

**eFigure.2 The missing rate of features extracted across MIMIC-IV databases.**

**
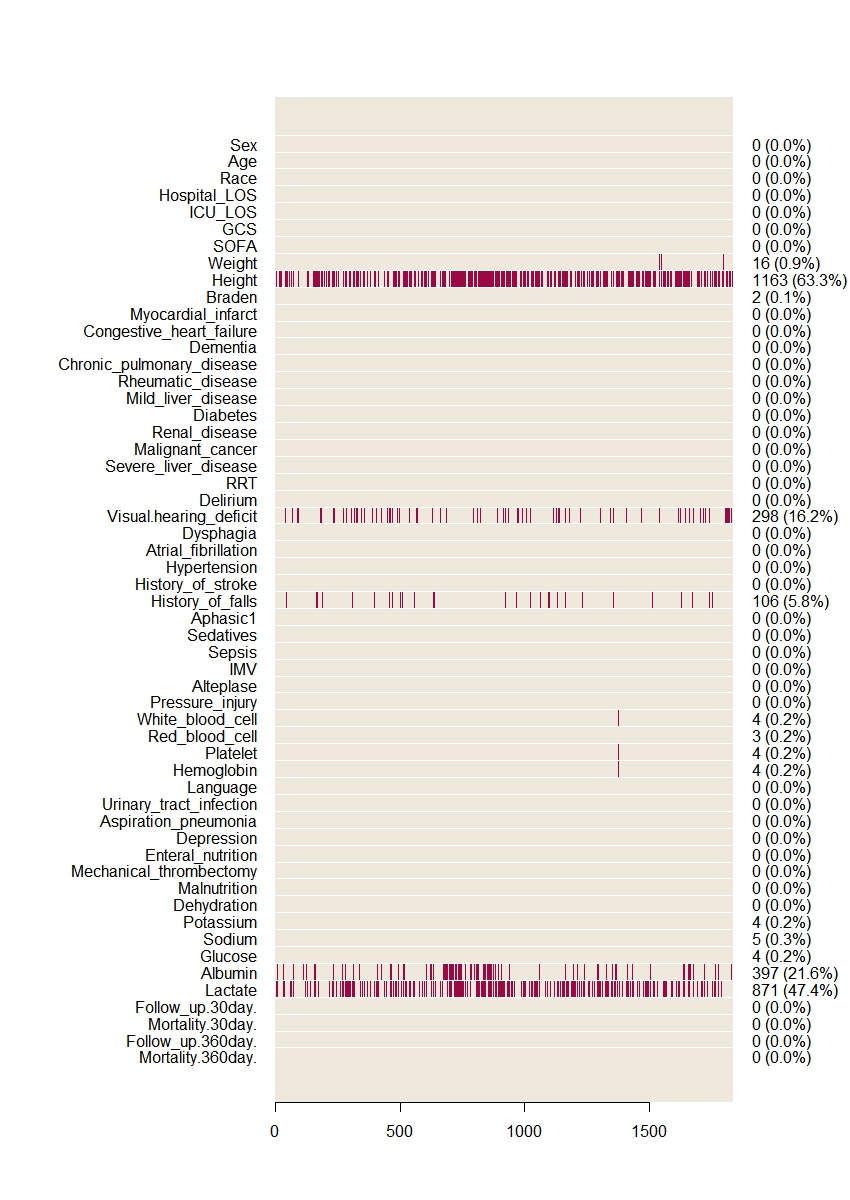
**

Abbreviations: MIMIC-IV: Medical Information Mart for Intensive Care IV.

Note: eFigure.2 Showed features missing rate from MIMIC-IV database; One red block represented one missing value for each feature.

**eFigure.3 Causal mediation analysis.**


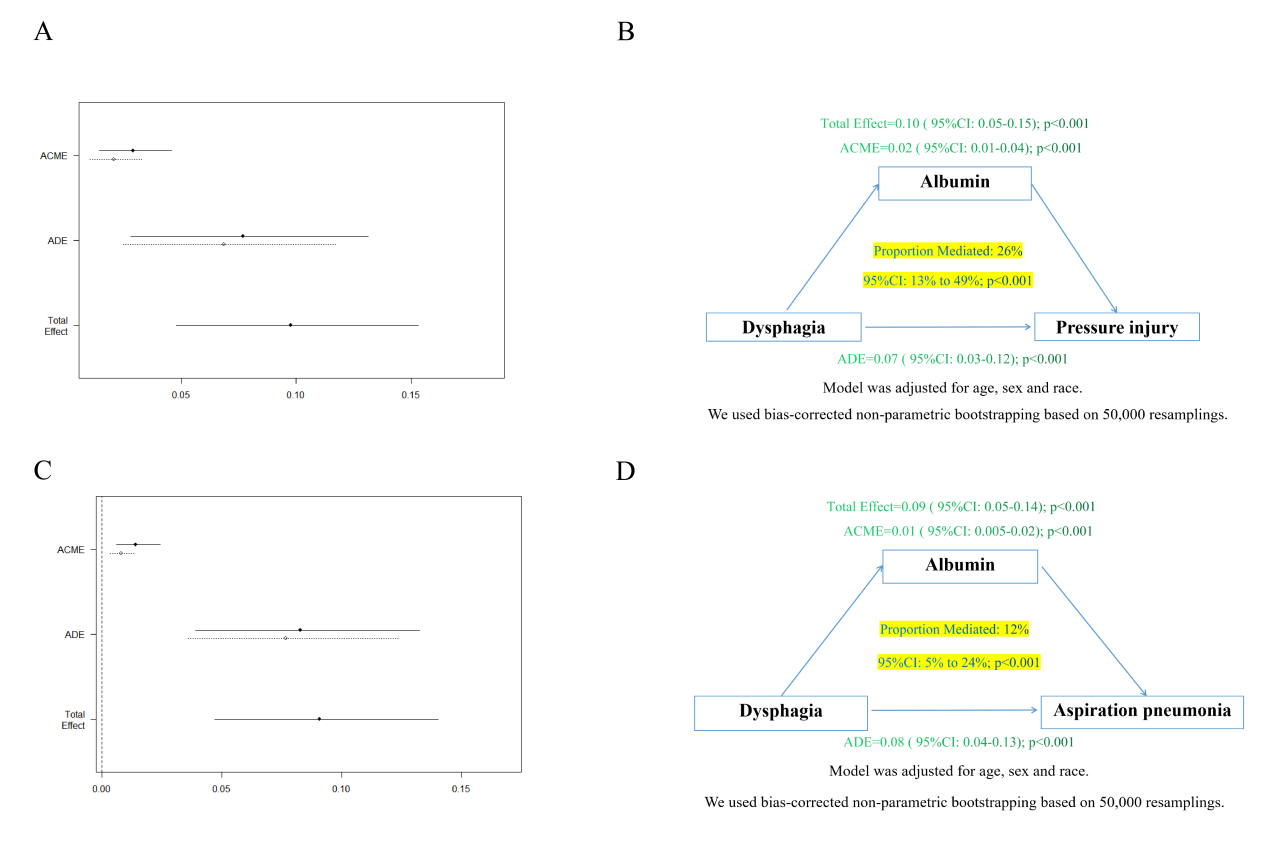


A)Process Diagram for Mediation Analysis (Pressure injury as a outcome)

B)Directed Acyclic Graph (Pressure injury as a outcome)

C)Process Diagram for Mediation Analysis (Aspiration pneumonia as a outcome)

D)Directed Acyclic Graph (Aspiration pneumonia as a outcome)

Note: Diagram showing components of mediation model testing effect of dysphagia lead on pressure injury/aspiration pneumonia mediated by serum albumin level.

**eFigure.4 Mediation analysis for the association between dysphagia and delirium.**


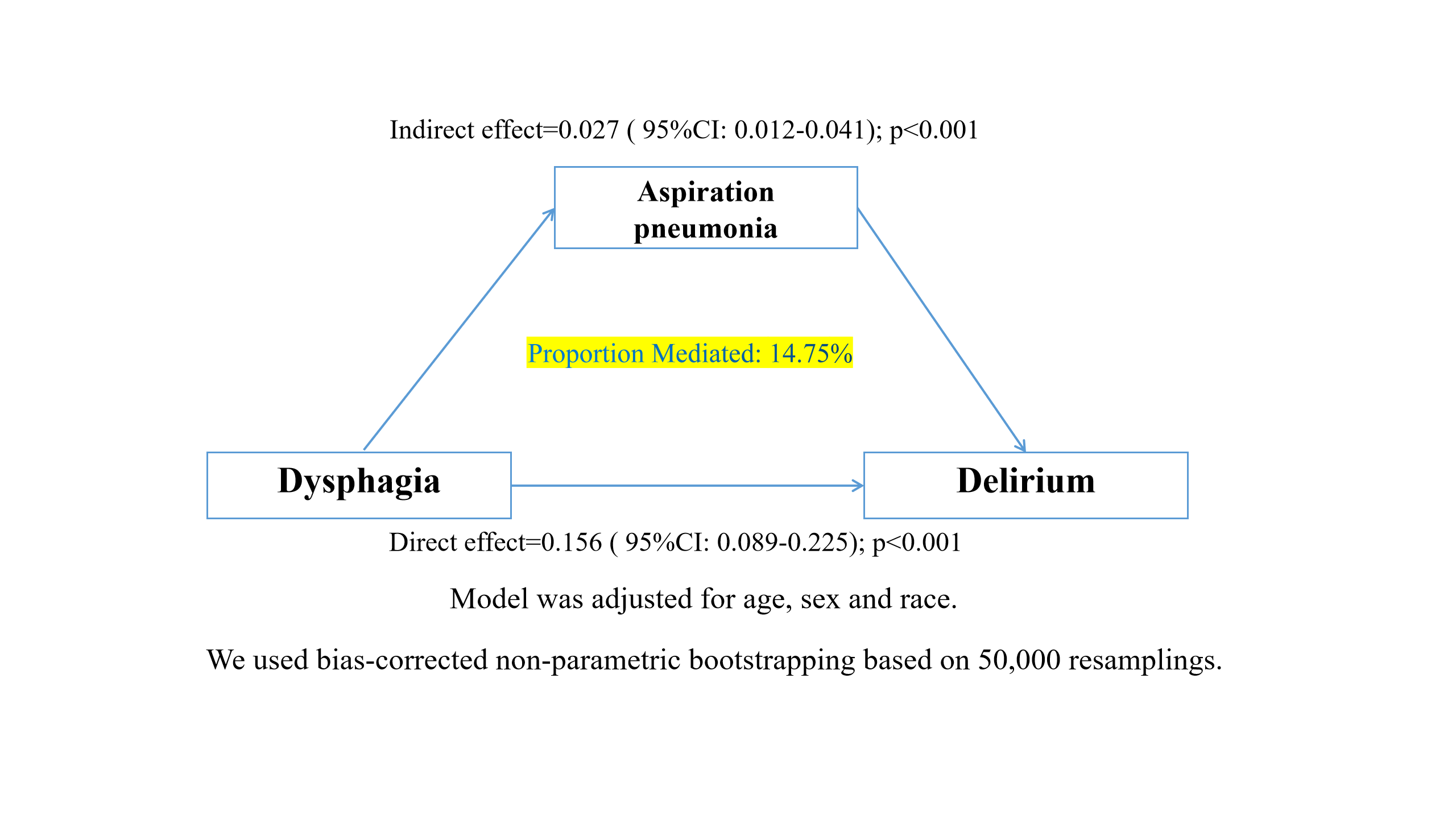


**eFigure.5 Standardized Mean Differences before and after Propensity Score Matching.**


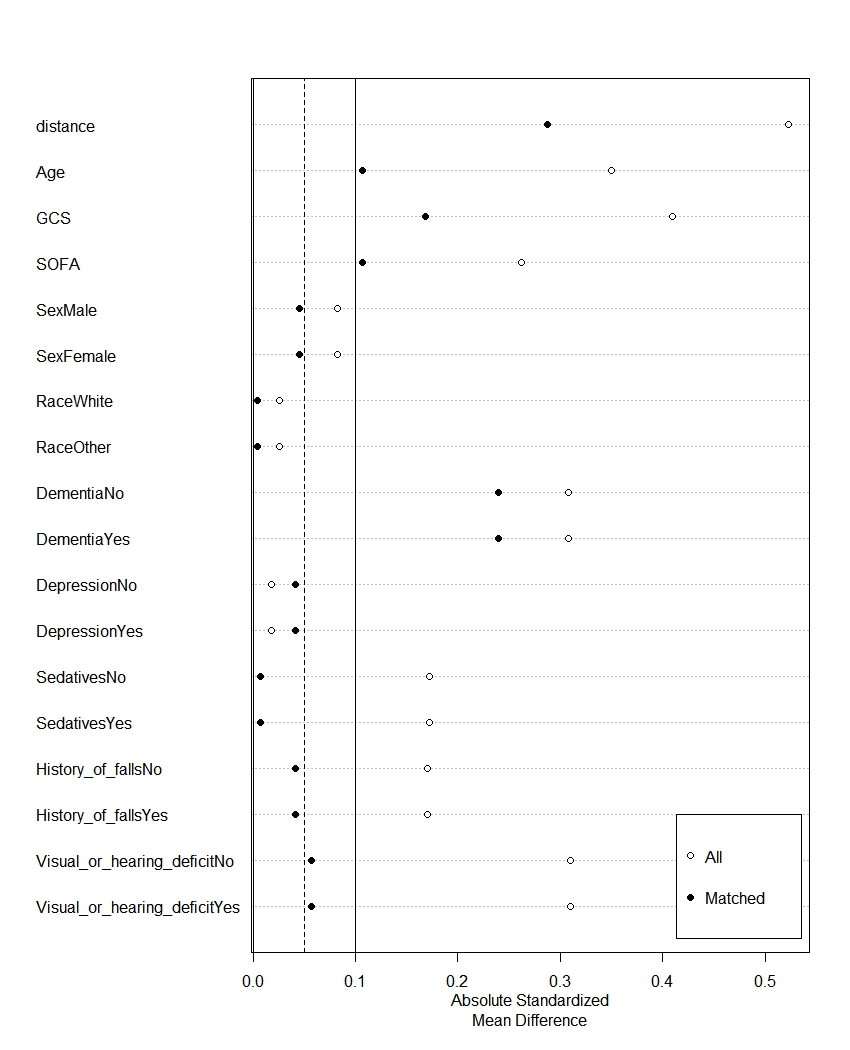


Abbreviations: GCS: Glasgow Coma Score; SOFA: Sequential Organ Failure Assessment.
